# Atypical cutaneous leishmaniasis: Contribution of confocal microscopy

**DOI:** 10.1016/j.jdcr.2023.11.034

**Published:** 2023-12-29

**Authors:** Meryem Soughi, Marco Ardigo, Saadia Boughaleb, Oumaima Zouine, Layla Tahri Elousrouti, Zakia Douhi, Sara Elloudi, Hanan Baybay, Fatima Zahra Mernissi

**Affiliations:** a Department of Dermatology, Hassan II University Hospital, Fez, Morocco; b URL CNRST N15, Human Pathology, Biomedicine and Environment Laboratory, Faculty of Medicine, Pharmacy and Dental medecine of Fez, Sidi Mohamed Ben Abdellah University, Fez, Morocco; c Dermatology Unit, IRCCS Humanitas Research Hospital, Rozzano, Italy; d Department of Biomedical Sciences, Humanitas University, Pieve Emanuele, Italy; e Biomedical and Translational Research Laboratory, Faculty of Medicine, Pharmacy, and Dental Medicine of Fez, Sidi Mohamed Ben Abdellah University, Fez, Morocco

**Keywords:** confocal microscopy, cutaneous leishmaniasis, Leishmania bodies

## Clinical presentation

A 17 years old patient, with no medical history and no recall of an insect bite, presented with a lesion on the nose progressively increasing in size during the last 3 months. The dermatologic examination disclosed an erythematous ulcerate tumor of 5 cm in diameter involving the tip of the nose. The diagnosis of leishmaniasis was suspected but the Giemsa stained smear examination for leishmania amastigotes was negative.

## Dermatoscopic appearance

**Fig 1:**
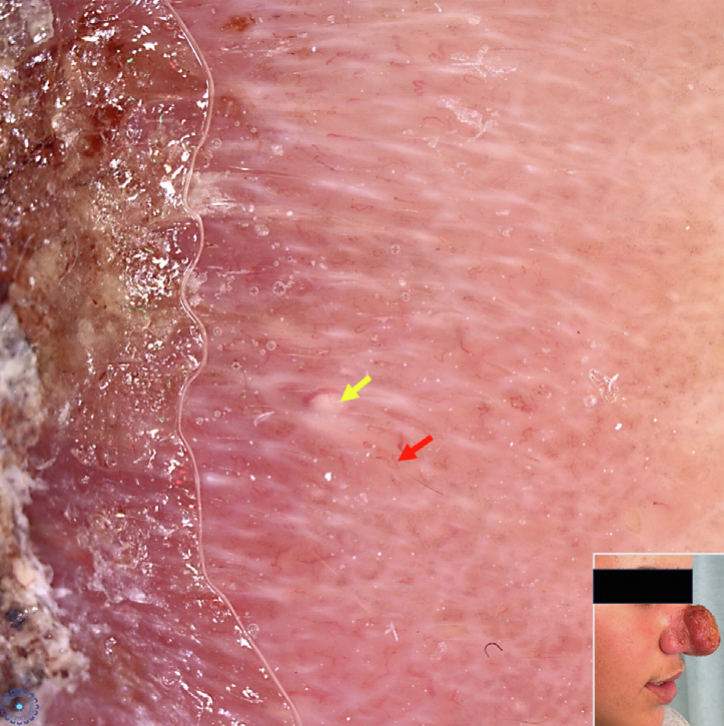
Dermatoscopic image of the edge of the nose tumor showing irregular comma shaped vessels (*red arrow*) and multiple yellowish teardrops (*yellow arrow*).

Dermatoscopy of the edges of the lesion showed comma vessels and yellow teardrops (Fig 1).

## Reflectance confocal microscopy appearance

**Fig 2:**
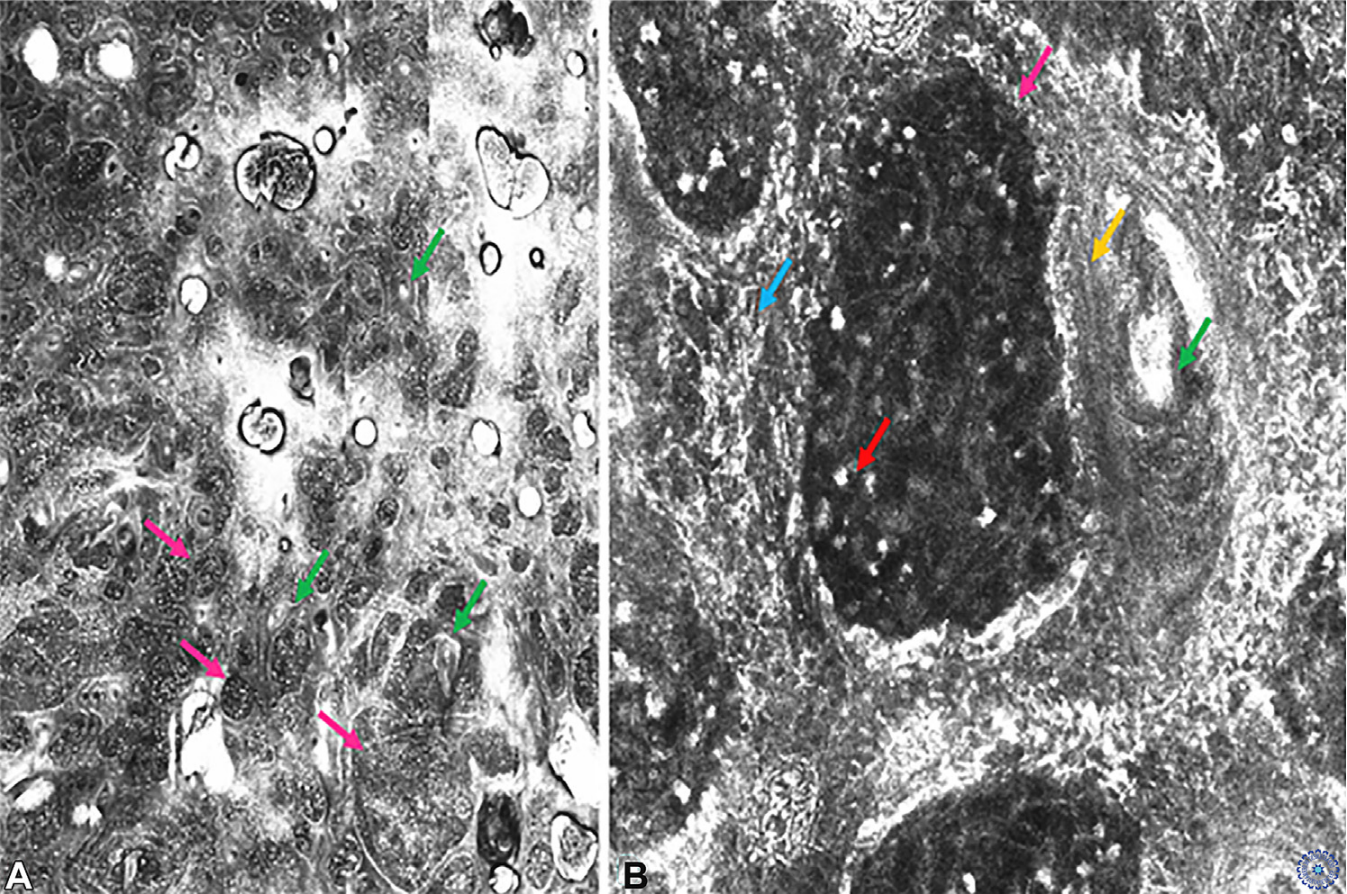
**A,** The papillary dermis shows structures resembling bird’s nests. These consist of brightly reflective interwoven fibers around hair follicles (*green arrows*) and granulomas (*pink arrows*). These look like follicles but are smaller and separated from the skin surface. **B,** Detail of reflectance confocal microscopy showing a “nest” made of intertwined dendritic Langerhans cells (*blue arrow*) and collagen bundles (*yellow arrow*) delimitating the granuloma “egg” (*pink arrow*) containing multiple bright round cells (*red arrow*) corresponding to leishmanian bodies, next to a hair follicle filled with keratin (*green arrow*) corresponding to the yellowish teardrop in dermoscopy.

Reflectance confocal microscopy (RCM) showed multiple inflammatory cells in the papillary dermis, associated with dendritic cells surrounding rounded nests; juxtaposed with the follicular plugs. The nests were delimited by intertwined hyperreflective fibers within which were found large refractile structures. Granulomas and follicles give the appearance of eggs in a bird’s nest (Fig 2).

## Histology

**Fig 3:**
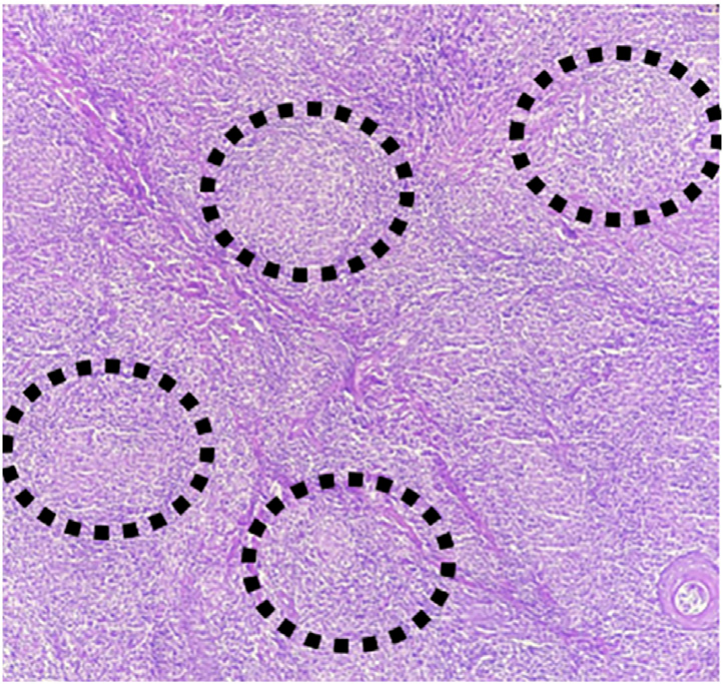
Histiocytic and epithelioid granulomas in the dermis (histological section, Hematoxylin-eosin Saffron (HES) stain; original magnification: ×200).

The histopathology showed epithelioid granulomas in the dermis (Fig 3).

**Fig 4:**
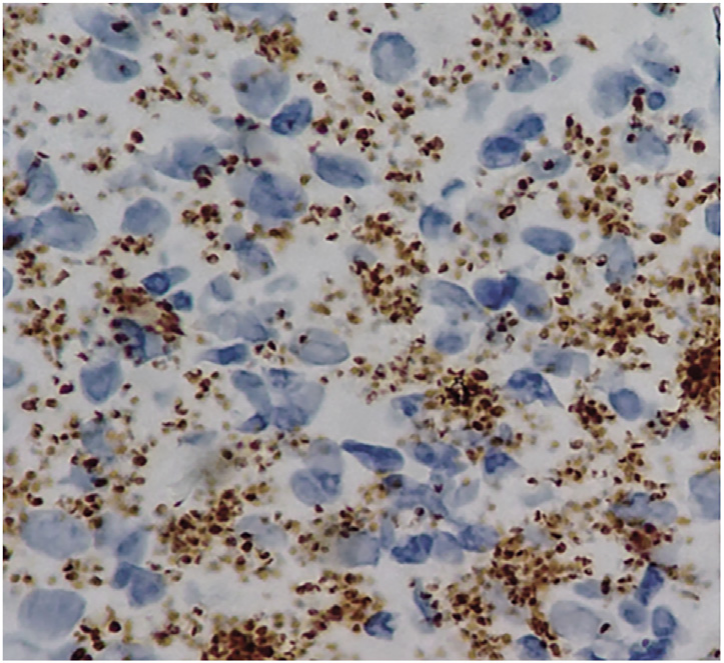
Immunohistochemistry ×1000 with anti-CD68 antibody which underlined numerous Leishmania bodies in the cytoplasm of histiocytic cells.

Immunohistochemistry showed a CD1a staining of dendritic cells, corresponding to a “nest” of Langerhans cells. The bright round cells were CD68^+^ (Fig 4), corresponding to histiocytes, including leishmania bodies. The genotyping of the cutaneous biopsy revealed *Leishmania tropica* species.

## Key message

Leishmaniasis is a tropical infection caused by an intracellular parasite of the genus *Leishmania*, transmitted to humans by sandflies.^1^ The diagnosis of cutaneous leishmaniasis is usually based on clinical features; however in some cases it is more difficult, especially in the case of an atypical form, such as the pseudotumor form as in our patient. It is confirmed through observing amastigotes in direct smears, culture, and histopathology or polymerase chain reaction, however, the biopsy sensitivity for diagnosing Old World cases of cutaneous leishmaniasis is 59%. On the other hand, visualizing amastigotes has a reported sensitivity range of 50% to 70%.^2^ In the case of our patient, no leishmania bodies were found either on the smear or histologically. Dermatoscopy has been proved to be a useful tool for the diagnosis. In our case it guided the diagnosis by showing comma vessels and yellow teardrops of the edge of the lesion, but, the smear for leishmania bodies was negative, which might be caused by a technical error. Only few cases of RCM of cutaneous leishmaniasis have been published. The features of RCM in our patient were strongly correlated with histopathology. The eggs in a bird’s nest has been described in one observation in the literature,^1^ corresponding to granulomas and follicles. We have also found dendritic cells corresponding to Langerhans cells and histiocytes, including leishmania bodies inside the “egg.” To our knowledge, only a single observation has reported leishmania bodies in confocal microscopy.^3^ This bodies seen within granulomas on RCM were the key feature in the initial diagnosis confirmed by histopathology.

Other features have been described especially multinucleated giant cells, vascular structures, such as curved vessels and brick-like structures. This could be *in vivo* imaging of partially degraded cornified material in the follicular plug.^4^

RCM remains a noninvasive imaging tool *in vivo* that can be used as a first diagnostic step before confirmations. We also suggest using it to monitor treatment, especially in cases that are resistant or respond poorly to treatment, by revealing leishmanial bodies and histiocytes within the inflammatory infiltrate.

## Conflicts of interest

None disclosed.
